# Five-Aminosalicylic Acid: An Update for the Reappraisal of an Old Drug

**DOI:** 10.1155/2015/456895

**Published:** 2015-01-21

**Authors:** Cristiana Perrotta, Paolo Pellegrino, Eliana Moroni, Clara De Palma, Davide Cervia, Piergiorgio Danelli, Emilio Clementi

**Affiliations:** ^1^Unit of Clinical Pharmacology, National Research Council-Institute of Neuroscience, Department of Biomedical and Clinical Sciences “Luigi Sacco”, University Hospital “Luigi Sacco”, Università di Milano, 20157 Milan, Italy; ^2^Unit of General Surgery, Department of Biomedical and Clinical Sciences “Luigi Sacco”, University Hospital “Luigi Sacco”, Università di Milano, 20157 Milan, Italy; ^3^Department for Innovation in Biological, Agro-Food and Forest Systems, Università della Tuscia, 01100 Viterbo, Italy; ^4^Scientific Institute, IRCCS Eugenio Medea, 23842 Bosisio Parini, Italy

## Abstract

Inflammatory bowel disease (IBD) comprises several conditions with chronic or recurring immune response and inflammation of the gastrointestinal apparatus, of which ulcerative colitis and Crohn's disease are the commonest forms. This disease has a significant prevalence and it is of an unknown aethiology. Five-aminosalicylic acid (5-ASA) and its derivatives are among the oldest drugs approved for the treatment of the IBD. In this review we reapprise aspects of 5-ASA mechanism of action, safety, and efficacy that in our opinion make it a valuable drug that can be fruitfully tailored in personalised treatments as a therapeutic option alongside other immune-modifying agents.

## 1. Introduction

Inflammatory bowel disease (IBD) is an idiopathic disease caused by a dysregulated immune response to host intestinal microflora and is comprised of two major disorders: ulcerative colitis (UC), which is limited to the colon, and Crohn's disease (CD), which can affect any segment of the gastrointestinal tract. Both are chronic inflammatory condition characterised by relapsing and remitting episodes of inflammation, which are transmural in CD and limited to the mucosal layer of the colon in UC [1, 2]. In the clinical practice, both UC and CD are typically classified as mild to moderate, moderate to severe, and severe to fulminant. Whereas the ultimate cause of IBD remains to be elucidated, a combination of several factors, such as genetic predisposition, environmental factors, and dysfunctions of the immune system, contributes significantly to the development of this disease. Patients with IBD are at increased risk for colorectal cancer (CRC) and should undergo specific screening with colonoscopy according to disease extent and duration [3].

The principal aims of the pharmacological treatment of IBD are inducing and maintaining the remission, obtaining mucosal repair, avoiding the surgical resection, and reducing the development of cancer as a result of chronic inflammation. The therapy of mild-to-moderate IBD depends on the location and degree of severity of the disease as well as patient acceptability and consists of oral or rectal therapies used individually or in combination. Severe or fulminant disease requires hospitalisation and intensive parenteral therapy [4].

Five-aminosalicylic acid (5-ASA) (mesalamine or mesalazine) and its derivatives (i.e., balsalazide, olsalazine, and sulfasalazine) are anti-inflammatory drugs indicated for the treatment of IBD. In particular, 5-ASA agents are the core therapy for UC and are recommended by the “American College of Gastroenterology, ulcerative colitis practice guidelines” for both induction and maintenance of remission in patients with mild-to-moderate UC [5]. More than 88% of all UC patients receive treatment with 5-ASA in USA as well as in Europe [6]. The use of 5-ASA drugs for CD remains instead controversial: a recent meta-analysis found that 5-ASAs taken as a group were superior to placebo for the induction of remission, but no significant benefit in remission rates was observed when only trials using 5-ASA were examined [7]. Additionally, no benefit was observed in the maintenance of remission compared with placebo [7].

## 2. Intracellular Mechanisms Coupled to 5-ASA Actions in Intestine Cells

A large body of experimental data shows that 5-ASA modulates multiple but not mutually exclusive pathways in the intestine [8, 9] where it supposedly acts locally on the mucosa. Since 5-ASA has a similar structure to acetyl-salicylic acid, it shares molecular targets, interfering with inflammation and proliferation, similar to those of aspirin and other nonsteroidal anti-inflammatory drugs.

Five-ASA was initially shown to downregulate the inducible cyclooxygenase/prostaglandin E2 (COX-2/PGE2) signalling in mucosa inflammatory cells [10, 11]. Later, 5-ASA targeting of COX-2 was also demonstrated in intestine cells. In particular, both basal and tumour necrosis factor (TNF-*α*)/interleukin- (IL-) 1*β*-induced COX-2 expressions are inhibited by 5-ASA thus reducing PGE2 synthesis [12], although mechanisms other than COX-2 modulation have also been suggested [12, 13]. Generally 5-ASA decreases the nuclear factor *κ*B (NF-*κ*B) activity induced by TNF-*α*, modulating the NF-*κ*B inhibitor, I*κ*B*α* [14–17], as well as the NF-*κ*B transcriptional activity induced by IL-1, although it does prevent neither IL-1-induced I*κ*B*α* degradation nor IL-1-induced nuclear translocation of NF-*κ*B family members [18].

Five-ASA was also found to increase *β*-catenin expression/phosphorylation and the expression of *μ*-protocadherin in intestine cells while reducing the expression of Wnt/*β*-catenin target genes and the activity of the protein phosphatase 2A [19–22].

More importantly, evidence is also accumulating suggesting a key role of peroxisome proliferator-activated receptor *γ* (PPAR-*γ*) in mediating the anti-inflammatory effects of 5-ASA. This transcription factor, belonging to the nuclear hormone receptor superfamily, modulates the inflammatory response of monocytes and macrophages by inhibiting the production of nitric oxide and macrophage-derived cytokines such as TNF-*α* and IL-1 and IL-6 [23]. In particular, 5-ASA enhances PPAR-*γ* expression/activity in intestine cells and promotes its translocation from the cytoplasm to the nucleus [22, 24, 25]. This is followed by the induction of the tumour suppressor gene PTEN, the activation of caspases 8 and 3, and the inhibition of antiapoptotic proteins [22, 25]. By promoting PPAR-*γ* nuclear translocation 5-ASA also reduces NF-*κ*B activation, increases the expression of I*κ*B*α*, restores STATs 1 and 3 protein concentrations, and reduces the expression of TNF-*α*, monocyte chemotactic protein-1, and the inducible nitric oxide synthase [17]. The importance of the 5-ASA to PPAR-*γ* signalling is clearly shown in experiments in which 5-ASA inhibition of growth of colon cancer xenografts is prevented by PPAR-*γ* antagonists [26]. Interestingly 5-ASA was found to normalise both PPAR-*γ* and heterodimer retinoid X receptor *α* expression after irradiation [17].

Despite the relevance of the 5-ASA to PPAR-*γ* signalling this pathway cannot explain all the effects of 5-ASA such that additional mechanisms have been proposed to be involved in the antiproliferative role of this drug [25]. Indeed, in intestine cells, 5-ASA also suppresses epidermal growth factor receptor phosphorylation/activation, likely through the modulation of the protein tyrosine phosphatases Src homology 2 [27]. In addition, the expression of *β*2-adrenoreceptor and *β*-arrestin 2 was found to be increased after 5-ASA treatment, suggesting an involvement of adrenergic receptors signalling in 5-ASA effects [16]. Recently, 5-ASA was shown to induce membranous expression of E-cadherin and increase cell adhesion through inhibition of p-21 activated kinase-1 and modulation of N-glycosylation [21, 28]. Finally, 5-ASA was found to interfere also with the mitogen activated protein kinase and phosphatidylinositol-4,5-bisphosphate 3-kinase/Akt pathways [21, 22] confirming further the pleiotropic mechanism of action of this drug.

## 3. Pharmacogenetics

Five-ASA is rapidly adsorbed and extensively acetylated to N-acetyl-5-ASA by the N-acetyltransferase 1 enzyme in intestinal epithelial cells and the liver [29]. Clinical evidence indicates the presence of a significant interindividual variability in terms of clinical response and safety to 5-ASA; it would be important to understand in full this variability to allow predicting patients who will either not respond or experience adverse events [7, 30, 31]. A hypothesis that may explain these clinical aspects is the presence of significant interindividual variability in drug acetylation. The first observation of this phenomenon was reported over 45 years ago, in patients treated with isoniazid for tuberculosis [32, 33] in which population were classified as slow and rapid acetylators [34], based on the genetic variation within the N-acetyltransferase genes (NAT) [34, 35].

Two isoenzymes, NAT1 and NAT2, have been identified in humans and share overlapping as well as specific aryl-amine substrates. The genes for both NAT1 and NAT2 are located on chromosome 8p22. Several variant alleles of the NAT1 gene have been described. A number of these are functional and result in a reduced N and/or O-acetylation (NAT ^*^14A, NAT1 ^*^14B, NAT1 ^*^17, NAT1 ^*^19, and NAT1 ^*^22) [36–38]. Eleven single nucleotide polymorphisms (SNPs) in the NAT2 gene result in 25 different alleles in humans [32, 34], several of which are associated with a slow acetylating phenotype. Such phenotype is common, with over 50% of Caucasian being slow acetylators [39]. Less frequent variants include the NAT2 ^*^12B, which is associated with high NAT2 expression and a fast acetylating phenotype [32, 35, 40].

The high frequencies of rapid and slow acetylators in the general population, alongside the potential effects of these SNPs on 5-ASA therapy, have suggested a possible explanation for the variability in drug safety and response observed in IBD patients [41]. Patients with a rapid acetylator genotype for NAT1 could potentially experience a decreased efficacy of 5-ASA, while an increased efficacy could potentially be observed in slow acetylators for NAT1.

SNPs within NAT genes may also play a role as risk factors for sulfasalazine toxicity. Sulfasalazine is a prodrug that is metabolised in the colonic lumen to 5-ASA and sulfapyridine. Sulfapyridine is responsible for most of the side effects that occur with sulfasalazine [39]. As sulfapyridine is inactivated in the liver by NAT2, slow acetylators could be at higher risk of toxicity [41]. Ricart and colleagues evaluated these concerns in an analysis on a cohort of patients with UC [41]. The analysis was carried out by enrolling 77 patients with UC and was aimed at evaluating the correlation between the most frequent polymorphisms for NAT1 and NAT2 (13 and 17, resp.) and clinical response to 5-ASA and sulfasalazine. The authors found no relationship between NAT1 genotype and response to 5-ASA or sulfasalazine. Additionally, no relationship between NAT2 genotype and toxicity to sulfasalazine was observed. Hausmann and colleagues achieved a similar conclusion by considering NAT1 genotype and clinical response in a cohort of 78 patients with UC [42]. These analyses indicate that, at variance with 5-ASA, NAT1/2 SNPs are unlikely to determine significant effects on 5-ASA and sulfasalazine therapy [39, 43–49].

## 4. Pharmacovigilance

Five-ASA is generally well tolerated and the most common side effects include headache, nausea, and abdominal pain [30]. Clinical trials showed that the proportion of patients treated with 5-ASA and experiencing adverse events was similar to the one observed in placebo-treated patients [31].

Watery diarrhoea occurs in up to 8% of patients taking oral preparations. Diarrhoea is usually mild and occurs at the initiation of therapy. Patients generally report that this condition resolves in 4 to 8 weeks, due to the ability of the colon to adapt to an elevated fluid load secondary to increased absorption [30, 31, 50].

Reports from postmarketing surveillance studies highlighted several cases of serious and nonserious adverse events temporally correlated with 5-ASA therapy, including nephrotoxicity, pancreatitis, respiratory failure, white cell aplasia, and myocarditis [51–59].

A French pharmacovigilance study of 5-ASA reported between 6.6 and 9.0 adverse events per million days of treatment, including cases of pancreatitis, hepatitis, haematological disturbances, and pericarditis [60]. Another study identified 393 and 514 adverse events per million prescriptions for 5-ASA and sulfasalazine, respectively, over an 8-year interval [61]. The odds ratio for any adverse event on sulfasalazine (relative to 5-ASA) was 1.31 (95% confidence interval, 1.22–1.40). However, this risk varied depending on the adverse event. Nephrotoxicity and pancreatitis were significantly more likely to be reported with 5-ASA, whereas haematological disturbances occurred more frequently with sulfasalazine [61]. These latter findings were criticised for several reasons and subsequent studies and meta-analyses found an adverse event profile similar to that of the placebo [62–64]. Finally, approximately 3% of patients on oral preparations have a paradoxical worsening of their colitis symptoms and should be considered allergic to 5-ASAs. Drug should be withdrawn and 5-ASAs should no longer be used [31].

### 4.1. Nephrotoxicity

Renal impairment represents one of the most debated adverse reactions attributed to 5-ASA [65–74]. A review of published clinical trials concluded that renal impairment might occur in up to one in 100 patients treated with this drug. Clinically significant impairment did not occur in any of the 500 patients for whom data were available. The incidence of clinically significant interstitial nephritis was estimated to be less than one in every 500 patients treated when serum creatinine is monitored regularly [75]. Yet, 5-ASA can cause interstitial nephritis. This includes reports of IBD patients in which renal function deteriorated during 5-ASA treatment and ameliorated after therapy discontinuation [75, 76]. A case of positive de- and rechallenge (i.e., a therapy discontinuation leading to improvement and therapy resuming leading to subsequent clinical deterioration) was also described [77]. The nephrotoxicity of 5-ASA was also shown in animal models [68]. The structural similarity of 5-ASA to drugs with well-documented nephrotoxic potential lends further support to the hypothesis that this drug initiates the interstitial inflammation in patients with IBD [68]. The absence of a clear relationship between 5-ASA dose and the risk of nephrotoxicity suggests that this complication is idiosyncratic [67]. A significant dilemma in the definition of causality association between 5-ASA and nephrotoxicity is because IBD itself can cause renal dysfunction. Patients with IBD may have extraintestinal manifestations of the disease involving the kidney, such as stones and amyloidosis. Additionally, nonspecific morphological changes in the glomeruli of patients with IBD may occur [78]. It has been calculated that approximately 10% of the patients with 5-ASA nephrotoxicity will develop end-stage renal disease, emphasising the need for timely recognition of renal impairment and prompt discontinuation of 5-ASA treatment of affected patients [5]. Thus, many authors agree on performing a monitoring of renal function by serum creatinine measurements, although the optimal schedule remains to be established and there is no evidence that such screening or monitoring improves patient outcomes [5]. A recent survey indicates that gastroenterologists tend to monitor renal function in IBD patients on 5-ASA, suggesting that this screening programme is widely accepted among clinicians [74].

### 4.2. Acute Pancreatitis

Acute pancreatitis (AP) is an inflammatory disease of the pancreas characterised by severe acute upper abdominal pain and elevated blood levels of serum amylase and/or lipase [79]. A variety of medications are listed among the aethiological factors for AP in the general population after gallstones and alcoholism [80–83]. The mechanisms of drug induced AP are not always clear but they have been proposed to include direct toxicity, hypersensitivity, secondary hyperlipidaemia, or hypercalcaemia [79]. More than one mechanism may be responsible for each individual case. While it is difficult to establish a causal role for some medications in the pathogenesis of AP [64, 84], several cases of AP following therapy with 5-ASA have been reported so far [85–90]. A suspected mechanism of such reaction was the similarity of the sulfonamide component to thiazide diuretics, an established cause of AP. However, with the use of 5-ASA medications, which lack the sulfonamide component, pancreatitis still occurs. Another interesting hypothesis to explain the mechanism of 5-ASA-induced pancreatitis is the local effect of 5-ASA that may increase pancreatic duct permeability [91]. Analysis of the reports received by the manufacturer and by the USA Food and Drug Administration adverse event report database indicates disproportionality in the number of reports for pancreatitis with Multi Matrix System and controlled-release 5-ASA [92]. Such disproportion suggests that the drug delivery mechanism is associated with variation of AP risk in IBD patient, although pharmacoepidemiological study to confirm this aspect formally is still lacking [92].

### 4.3. Myocarditis

Classic myocarditis refers to an inflammation of the heart muscle caused by a variety of triggers including infections, drugs, and autoimmune diseases [93]. Cardiac disease may develop in Crohn's patients either as a disease extraintestinal manifestation or as an adverse reaction to 5-ASA therapy. Such occurrence is well reported and documented, with several cases described in literature reports [54, 94–100]. 5-ASA-associated myocarditis is a rare but potentially life-threatening manifestation, generally occurring 2–4 weeks after the initial exposure to the drug [94]. However, the time between the first exposure and myocarditis occurrence might be delayed, especially in case of concomitant steroids therapy [97, 98]. Drugs can determinate myocarditis as a direct toxic agent or by inducing a hypersensitivity reaction [101]. The mechanism by which 5-ASA can trigger myocarditis is likely to be a humoural-mediated hypersensitivity reaction [101], thus similar to the one already described for penicillin and tricyclic antidepressants [97, 99, 102]. Several recent findings support this hypothesis: first, inflammation worsens if 5-ASA is reintroduced during the acute phase; second the symptom resolution occurs within one week after drug discontinuation; third myocardium biopsy shows eosinophilic infiltration [95, 96, 98, 103].

### 4.4. Adherence

Adherence to 5-ASA treatment represents an important source of concern. According to reports from clinical trials in IBD patient, adherence rates range between 70% and 95%. In the normal clinical practice, however, nonadherence rates (defined as taking less than 80% of prescribed medication) range between 40% and 72% [104]. Adherence is a composite and multifactorial issue, in which a variety of factors play a role, including lack of insight into illness, perceptions and beliefs about the illness, treatment of asymptomatic disease, forgetfulness, and, last but not least, poor physician-patient relationship. A cohort study of 99 UC patients in remission demonstrated a considerable impact of adherence on clinical recurrence, with a fivefold greater risk of recurrence in nonadherent patients [104].

## 5. Five-ASA Shadows and Lights

Thirty years have passed since 5-ASA (in the form of sulfasalazine) was introduced into the management of IBD, and today it remains the backbone of treatment of UC for achieving and maintaining remission [5]. From the time of the launch onto the market until now many efforts have been made to develop new 5-ASA formulation acting, on the one hand, to improve its release in the colon by oral formulations and, on the other, to reduce the dosing schedule from a multiple- to a once-daily dosing [5]. The optimal number of 5-ASA doses per day for treating mild-to-moderate UC is still an area of some discussion. The use of multiple daily dosing (4 times per day) was at first based on the pharmacokinetics of sulfasalazine, where peaks and troughs of drug plasma levels occurred quickly. However, upon sulfasalazine administration, 30% of patients experienced adverse events and 50% stopped medical therapy [105]. Currently, several 5-ASA formulations are designed with once-daily administration, such as 5-ASA-Multi Matrix System, which is used for induction therapy, and prolonged-release 5-ASA granules, which are approved for the treatment of acute episodes and the maintenance of remission [106]. Systemic concentration measurements, which allow understanding the drug's distribution within the body, are used for a variety of drugs to estimate therapeutic effects. However, a series of 5-ASA peculiarities may impair the efficacy of this type of diagnostic test. 5-ASA acts topically and only low levels are released into the plasma via the colonic mucosa, thus making it difficult to relate mucosal and plasma concentrations. In their recent review on 5-ASA formulations used for UC therapies, Lichtenstein et al. pointed out that mucosal biopsies could be used to establish colonic distribution and to predict clinical efficacy [107]. The association between high mucosal 5-ASA levels and low disease activity has been shown in several studies, and interindividual variability in these levels has been proposed to be a useful tool to explain the differences observed in clinical effectiveness [108, 109]. A relationship between dose and response has been analysed recently in two systematic reviews from the Cochrane database both in induction and maintenance of remission therapies [64, 110]. Oral 5-ASA administered once daily has been found to be as effective as conventional dosing (twice or three times daily) for induction in mild-to-moderately active UC and for maintenance of remission in quiescent disease state. In patients with mild UC a dosage of 4 to 4.8 g/day does not appear to provide any additional benefit over a dosage of 2 to 2.4 g/day. However, patients with severe symptoms and moderate UC may benefit from an initial dosage of 4 to 4.8 g/day [110]. Recently, the Assessing the Safety and Clinical Efficacy of a New Dose of 5-ASA (ASCEND) III study suggested the advantage of the 4.8 g/day dose for patients affected by moderate disease treated previously with corticosteroids, oral mesalamines at low doses, rectal therapies, or multiple UC medications [111]. Few dose ranging studies for maintenance therapy have been performed so far, thus limiting the conclusions that can be drawn. In general it appears that patients with extensive UC or with frequent relapses may benefit from a higher dose of maintenance therapy [64]. Further studies are required to elucidate fully this topic.

Another important aspect among 5-ASA indications that is subject of debate is the chemoprotective role of the drug against the CRC as a consequence of IBD. Preclinical* in vitro* studies have shown that 5-ASA has antineoplastic properties by reducing inflammation and oxidative stress, inhibiting cell proliferation and promoting cell death, thus indicating that 5-ASA may not only act on the inflammatory cells, but also target cancer cells and inhibit pathways that are involved in sporadic CRC cell growth and survival [8, 9, 112]. The first report showing a reduction in CRC risk associated with 5-ASA use in UC patients was published in 1994 [113]. Since then, several observational studies have reported contradictory data about the chemoprotective action of 5-ASA; unfortunately, even data coming from meta-analyses are difficult to interpret because of the selection bias and the heterogeneity among the studies analysed [114–116]. Thereby, while the favourable effects of 5-ASA on CRC prevention are biologically conceivable and validated* in vitro*, the clinical confirmation of the relationship between the use of 5-ASA and the reduction of the risk CRC is still insufficient and requires further investigation.

## 6. Outlook on 5-ASA in Paediatry

A large number of preclinical and clinical studies testify the continuous attention on 5-ASA and on finding new therapeutic indications. In this respect, the possibility to use 5-ASA in the treatment of IBD in the paediatric population is a relevant issue [117]. The main problem in the treatment of paediatric patients is the lack of information on the paediatric use of most drugs that prevent often the optimal treatment of the patients. This occurs in the treatment of paediatric IBD, where few of the therapies used to induce and maintain its remission have been studied in paediatric clinical trials, to the point that most of information has had to be extrapolated from adult data [117]. Children need individualised therapies that take several factors into consideration including the child's age and size and the specific disease manifestations such as location of inflammation, duration, and prior response to therapy. Drug dosages also must be adjusted, based upon the child's pharmacodynamic parameters. For the treatment of mild-to-moderate UC and CD in children, in most cases 5-ASA is the initial therapy [117]. The strength of the administration to children of 5-ASA* versus* other treatments available lies in its low risk profile although the safety of 5-ASA, which is well established in the adult population, lacks confirmation in children. In addition, a number of issues remain to be solved. The dosage for children is extrapolated on a per-kilogram basis from data in adults. The number of treatments required per day and the frequency of administration for achieving efficacy (3-4 times per day) make compliance with 5-ASA very difficult in paediatric patients. A recent study conducted in clinical practice centres across North America and Europe reported that oral, delayed-release 5-ASA is an effective treatment in children with mild-to-moderate UC treated for 6 weeks [118]. Of notice, low and high doses of 5-ASA were similarly effective and well tolerated, with only few patients discontinuing treatment owing to an adverse effect [118]. Another study showed that a daily treatment of a 5-ASA is safe and efficacious in children and adolescents with ulcerative proctitis, the initial manifestation in a large portion of newly diagnosed UC cases [119]. Among the clinical trials found on https://clinicaltrial.gov/ (a service of the USA National Institute of Health) that have been proposed and conducted in the paediatric population to construct a model for an individualised 5-ASA treatment to improve the current outcomes, notable is the “Predicting Response to Standardized Pediatric Colitis Therapy” (PROTECT) study. This study is still ongoing and intends to evaluate the outcome of a best practices protocol (5-ASA alone and in combination with the corticosteroid prednisone) for the treatment of children newly diagnosed with UC. This study has as primary outcome the remission of the disease after one year of treatment. Moreover, it aims to stratify patients based on clinical, genetic, and immunologic tests in order to determine the impact of these parameters on the primary outcome and to understand the interpatients variability in response to therapy. The elucidation of the reason of interpatient variability in the response to therapy that should be achieved through the PROTECT study, as well as other studies still currently in progress, may open new perspectives, with the aim to maximise the benefit of 5-ASA therapy in children thus reducing the administration of corticosteroids, more potent but also more toxic, and/or the need for colectomy.

## 7. Conclusion

In several countries, IBD has been increasing in incidence and prevalence with the highest rates in USA, Canada, and Europe [120]. Despite recent therapeutic advances, morbidity is still high and may affect patient's quality of life. Five-ASA is the first line treatment of patients with UC from mild to moderate, used with the intent to induce remission, promote mucosal healing, and minimise steroid use and its toxicity. Unfortunately, about 30% of patients treated with 5-ASA are considered not responders, because of allergy, intolerance, underdosage, refractoriness to the treatment, and the need of switching their therapy to more potent and toxic medications. New strategies are under investigation to avoid this issue. For example, even in the absence of any prospective study, combination therapy of different formulation of 5-ASA (oral and topical) may be considered in patients who fail to respond to the monotherapy before switching to steroids. The safety profile is indeed one of the strengths of 5-ASA and may account for its usage in specific populations. In this regard new clinical trials have been carried out on these patients and the results may shed light on optimising 5-ASA therapy.
